# Profiling pro-neural to mesenchymal transition identifies a lncRNA signature in glioma

**DOI:** 10.1186/s12967-020-02552-0

**Published:** 2020-10-07

**Authors:** Qingyu Liang, Gefei Guan, Xue Li, Chunmi Wei, Jianqi Wu, Peng Cheng, Anhua Wu, Wen Cheng

**Affiliations:** 1 Department of Neurosurgery, The First Hospital of China Medical University, Nanjing Street 155, Heping District, Shenyang, 110001 Liaoning China; 2 Department of Radiotherapy, The Affiliated Tumor Hospital of Guangxi Medical University, Nanning, Guangxi China

**Keywords:** Glioma, PMT, lncRNA, Prognosis, ceRNA network, Immune

## Abstract

**Background:**

Molecular classification has laid the framework for exploring glioma biology and treatment strategies. Pro-neural to mesenchymal transition (PMT) of glioma is known to be associated with aggressive phenotypes, unfavorable prognosis, and treatment resistance. Recent studies have highlighted that long non-coding RNAs (lncRNAs) are key mediators in cancer mesenchymal transition. However, the relationship between lncRNAs and PMT in glioma has not been systematically investigated.

**Methods:**

Gene expression profiles from The Cancer Genome Atlas (TCGA), the Chinese Glioma Genome Atlas (CGGA), GSE16011, and Rembrandt with available clinical and genomic information were used for analyses. Bioinformatics methods such as weighted gene co-expression network analysis (WGCNA), gene set enrichment analysis (GSEA), Cox analysis, and least absolute shrinkage and selection operator (LASSO) analysis were performed.

**Results:**

According to PMT scores, we confirmed that PMT status was positively associated with risky behaviors and poor prognosis in glioma. The 149 PMT-related lncRNAs were identified by WGCNA analysis, among which 10 (LINC01057, TP73-AS1, AP000695.4, LINC01503, CRNDE, OSMR-AS1, SNHG18, AC145343.2, RP11-25K21.6, RP11-38L15.2) with significant prognostic value were further screened to construct a PMT-related lncRNA risk signature, which could divide cases into two groups with distinct prognoses. Multivariate Cox regression analyses indicated that the signature was an independent prognostic factor for high-grade glioma. High-risk cases were more likely to be classified as the mesenchymal subtype, which confers enhanced immunosuppressive status by recruiting macrophages, neutrophils, and regulatory T cells. Moreover, six lncRNAs of the signature could act as competing endogenous RNAs to promote PMT in glioblastoma.

**Conclusions:**

We profiled PMT status in glioma and established a PMT-related 10-lncRNA signature for glioma that could independently predict glioma survival and trigger PMT, which enhanced immunosuppression.

## Background

Glioma is the most common primary brain tumor, and despite great improvements in the treatment modalities for glioma, including surgery, radiotherapy and chemotherapy, patients with glioma, especially glioblastoma multiforme (GBM), still have unfavorable outcomes [1, 2]. To explore more effective treatments, molecular subtypes related to GBM prognosis have been identified based on large genomic data. Recently, GBM was classified into four molecular subtypes: pro-neural (PN), neural (NL), classical (CL), and mesenchymal (MES) [3]. Transition among the subtypes often occurs during gliomagenesis, which induces biological heterogeneity, poor prognosis, and therapeutic resistance [4, 5]. Among the four molecular subtypes, glioma cells cultured in vitro are mostly classified into PN or MES and show distinct biological features [4]. Compared with the PN subtype, the MES subtype is associated with aggressive phenotypes and poor prognosis [6]. Additionally, remnant gliomas after radiotherapy and/or chemotherapy can undergo pro-neural to mesenchymal transition (PMT), which is associated with treatment resistance [7]. Therefore, uncovering the mechanisms that underlie PMT is urgently needed to improve glioma treatments.

Long non-coding (lnc)RNAs are non-coding RNAs of greater than 200 bp in length. Previous studies have highlighted the important roles of lncRNAs in tumorigenesis and risky progression. Various lncRNAs have been found to be abnormally expressed in tumors, and thus are considered potential molecular targets [8]. Recently, several lncRNAs were noted to promote PMT in GBM. For example, blocking the lncRNA MIR155HG axis with the small molecule inhibitor NSC141562 suppressed mesenchymal transition [9]. Liu et al. [10] found that high LINC00152 expression might trigger PMT through the NF-κB pathway in GBM. However, a systematic understanding of how lncRNAs contribute to PMT is lacking for glioma. Hence, there is an urgent need to screen out PMT-related lncRNAs and clarify their potential mechanisms.

In this study, public glioma transcriptomic data from The Cancer Genome Atlas (TCGA), the Chinese Glioma Genome Atlas (CGGA), Rembrandt, and GSE16011 were collected for systemic analyses. We first calculated PMT scores with the single-sample gene set enrichment analysis (ssGSEA) algorithm, which reflects the PMT balance in glioma. Through this analysis, we found that PMT scores were positively associated with risky behaviors in glioma. We further identified PMT-related lncRNAs by weighted gene co-expression network analysis (WGCNA), and established a PMT-related 10-lncRNA risk signature by univariate Cox and least absolute shrinkage and selection operator (LASSO) analyses. The PMT-related lncRNA signature could independently predict the prognosis in high-grade glioma, and stimulate PMT processes to reprogram the immune microenvironment through a competing endogenous (ce)RNA network in GBM. Therefore, these findings will contribute to a deeper understanding of the mechanism of PMT, and highlight the potential application of the 10 PMT-related lncRNAs in glioma treatment strategies.

## Materials and methods

### Patient datasets and clinical information

LncRNA expression data from glioma patients was acquired from The Atlas of Noncoding RNAs in Cancer (TANRIC) [11], which was retrieved from TCGA glioma RNA-seq database (https://cancergenome.nih.gov/) and RNA-seq files from other independent studies in the CGGA database [12]. Coding mRNA expression profiles of RNAseq or microarray were collected from TCGA, CGGA, GSE16011, and Rembrandt. After acquiring the data, we annotated all samples according to their barcode ID based on available clinical information from the UCSC Xena (https://xenabrowser.net/datapages/) and CGGA (https://www.cgga.org.cn) databases. The glioma patients with detailed clinical and molecular information are described in Additional file 1: Table S1. We also downloaded matched miRNA microarray data from TCGA database.

### Establishing the PMT-related lncRNA signature

To establish the PMT-related lncRNA signature, we first collected PN (M2115) and MES (M2122) gene sets from the GSEA database (https://www.gsea-msigdb.org/gsea/index.jsp), and then calculated PN and MES scores using the ssGSEA algorithm of the GSVA package. PMT scores were generated by subtracting the PN score from the MES score. LncRNA expression profiles from TCGA RNAseq were selected as the training set. We first screened out differentially expressed lncRNAs (DELs; |logFC| > 1, P < 0.05) in GBM compared with non-GBM (oligodendroglioma, oligoastrocytoma, and astrocytoma) by the Limma package. Then the DELs were used for WGCNA analysis to identify lncRNA co-expressed modules, and to further determine PMT-related lncRNAs with both a gene significance (GS) > 0.5 and a module membership (MM) > 0.7, as previously described [13]. PMT-related lncRNAs were used for further univariate Cox regression analyses in all glioma, non-GBM, and GBM cohorts. We identified 17 lncRNAs that significantly predicted survival in all three cohorts, and these were then used to conduct further LASSO and Cox regression analyses. The selected criteria and PMT-related lncRNA risk scores were calculated using a formula described by Chai et al. [14]. The coefficient was also used to calculate a risk score for each case in the validation dataset (CGGA).

### Screening out signature-related genes (SRGs) in GBM from TCGA and CGGA

Pearson’s correlation analysis was performed to assess the relationship between coding mRNA expression and risk score in GBM. The SRGs (R > 0.3, P < 0.05) were selected for Gene Ontology (GO) analysis with the ClueGO plug-in in Cytoscape [15].

### Constructing the PMT-related lncRNA/miRNA/mRNA network

First, predicted binding of micro (mi)RNAs with the 10 lncRNAs were collected using the DIANA tools and LncBase Predicted v.2 (https://www.microrna.gr/LncBase/). LncRNA/miRNA pairs and potential target genes of overlapping miRNAs were generated as described in our previous study [16]. Then, we identified miRNA-mRNA pairs by intersecting target genes with SRGs. Finally, we constructed the lncRNA/miRNA/mRNA networks with Cytoscape.

### Cell transfection, qRT-PCR, protein isolation and western blotting

siRNAs for LINC01503 suppression were synthesized by GenePharma (Suzhou, China) and had the following sequences: si-LINC01503-1 sense, 5′-GGAGACAAAUGACGGCCUUTT-3′; si-LINC01503-2 sense, 5′-GGAGAAAGUUCUUUCCCUGTT-3′; and si-LINC01503-3 sense, 5′-GGACGAAUGCAGAGCCCUATT-3′. The effect of LINC01503 suppression was validated by qRT-PCR described in previous study [16]. The primer sequences were as the follows: LINC01503 (forward primer: GGGGACGGAGACAAATGAC, reverse primer: CACACTTGTCAGAGGCGTTC); CD44 (forward primer: CTGCCGCTTTGCAGGTGTA, reverse primer: CATTGTGGGCAAGGTGCTATT); OLIG2 (forward primer: TGGCTTCAAGTCATCCTCGTC, reverse primer: ATGGCGATGTTGAGGTCGTG); 18S (forward primer: GCAGAATCCACGCCAGTACAAGAT, reverse primer: TCTTCTTCAGTCGCTCCAGGTCTT). The RNA expressions of LINC01503, CD44, and OLIG2 were calculated by the 2^−ΔΔCt^ method and normalized to 18S mRNA expression. The procedure of protein isolation and western blotting was similar as that described in previous study [16]. The primary antibodies were as follows: CD44 (1:1000; Cell Signaling Technology, Boston, USA), OLIG2 (1:1000; Abcam, Cambridge, UK), β-actin (1:1000; Proteintech, Rosemont, IL, USA).

### Statistical analysis

Further enrichment analysis of the biological functions between two groups was performed by GSEA (https://software.broadinstitute.org/gsea/index.jsp). Scores of related gene sets were calculated by ssGSEA. A protein–protein interaction (PPI) network was constructed in Cytoscape. Hub genes were generated as previously described [16]. Based on the median score, glioma cases were divided into low- and high-score groups. Differences between the two groups were estimated with the Welch *t*-test, Wilcoxon rank sum test, or χ^2^ test. Kaplan–Meier survival curves were evaluated with the log-rank test. Univariate and multivariate Cox regression analyses were performed to identify independent prognostic factors. A two-sided P value < 0.05 was considered statistically significant.

## Results

### PMT scores were positively associated with the risky behaviors of gliomas

**Fig. 1:**
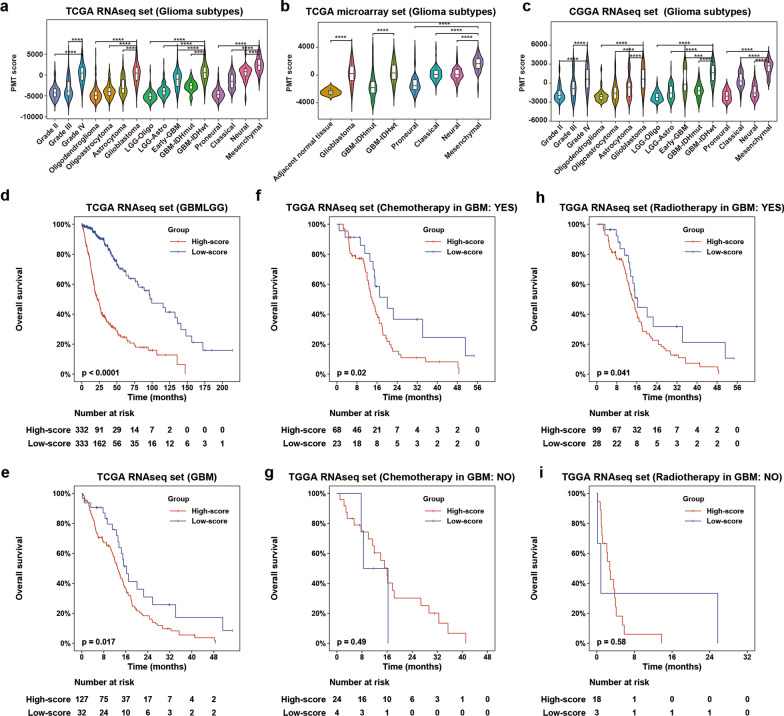
Distribution and prognostic value of PMT scores in glioma. **a**–**c** Distribution of PMT scores according to WHO grade, histopathological type, TCGA subtype, and molecular subtype in TCGA datasets and the CGGA RNAseq set. ****P < 0.0001. **d**, **e** Prognostic value of PMT scores in all glioma cases and GBM. **f**–**i** Prognostic value of PMT scores in different cohorts of GBM stratified by chemotherapy or radiotherapy in TCGA RNAseq set

To explore the general pattern of PMT status in glioma, we compared the distribution of PMT scores according to WHO grade, histology, and molecular subtype. We found that PMT scores were increased with increased risky progression of gliomas. Cases with higher grades from TCGA RNAseq dataset exhibited higher PMT scores (Fig. 1a). According to histopathologic classification, cases with higher PMT scores were enriched in order of GBM, astrocytoma, oligoastrocytoma, and oligodendroglioma. Regarding the five molecular subtypes described in our previous study [17], PMT scores were higher in the early-GBM (LGG-IDH-wt) and GBM with wild-type IDH (GBM-IDHwt, Fig. 1a). Similar PMT score distributions were verified in TCGA microarray, CGGA RNAseq, and microarray, Rembrandt, and GSE16011 datasets (Fig. 1b, c, Additional file 2: Fig. S1A–D).

According to TCGA microarray gene expression profiles, the gliomas were classified into four subtypes [3]. The MES subtype had the riskiest progression and most unfavorable survival prognosis. Here, we found that PMT score was significantly higher in the MES subtype than in the PN subtype in multiple public glioma datasets (Fig. 1a–c, Additional file 2: Fig. S1B–D). Meanwhile, glioma cases, even GBM, with higher PMT scores had poorer survival prognoses (Fig. 1d, e, Additional file 2: Fig. S1E–M). Furthermore, there was no difference in survival between the cases with low and high PMT scores without radiotherapy or chemotherapy. However, cases with higher PMT scores that received radiotherapy or chemotherapy still suffered reduced survival time (Fig. 1f–i, Additional file 2: Fig. S2). These results suggested that the PMT balance plays a critical role in the risky progression of glioma.

### Identification of PMT related lncRNAs by WGCNA analysis

**Fig. 2:**
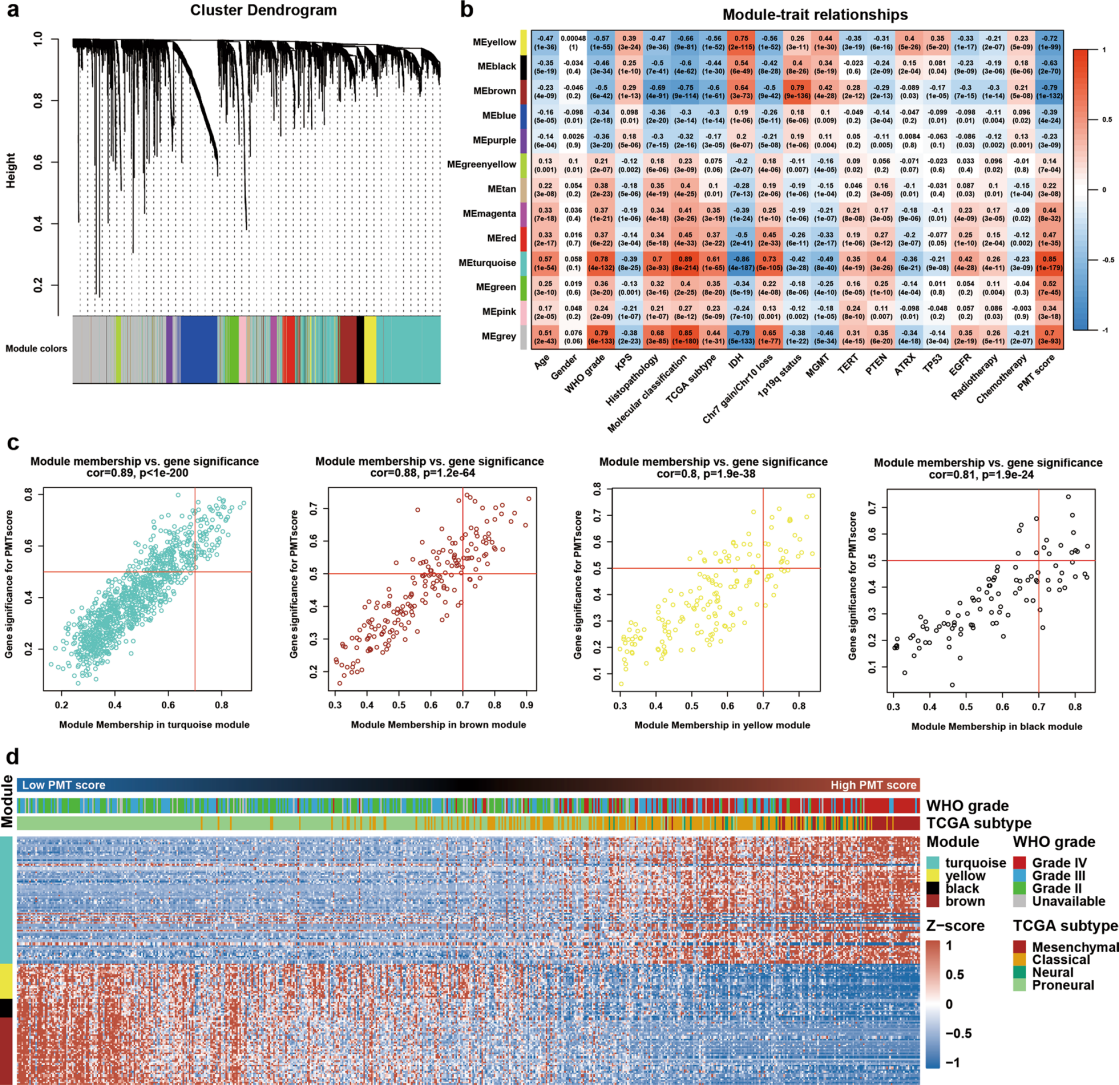
Identification of PMT-related lncRNAs by WGCNA. **a** Construction of co-expression lncRNA modules in the training set (TCGA). The branches of the cluster dendrogram refer to the 12 co-expression modules. **b** Pearson’s analysis between lncRNA modules and clinical and molecular features was performed. The color of each cell was varied according to the correlation coefficient. Relative P-values and R values were also annotated. **c** Correlation between gene significance (GS) and module membership (MM) in the turquoise, brown, yellow, and black modules was performed by scatter plot analysis. **d** In total, 149 PMT-related lncRNA expression profiles were shown by heatmap according to increasing PMT score

To determine the PMT-related lncRNAs with potential biological functions, we conducted WGCNA analysis to generate lncRNA co-expression modules. After removing outlier cases (Additional file 2: Fig. S3A and B), 3308 DELs were divided into different modules by WGCNA cluster analysis in the training set (TCGA RNAseq set, Fig. 2a). Here, we chose β = 4 as the soft threshold to build a scale-free network (Additional file 2: Fig. S3C), and then obtained 12 lncRNA co-expression modules for further analyses (Fig. 2a). All non-co-expression lncRNAs were gathered into the grey module (Fig. 2a).

To assess correlations between each module and PMT score, overall lncRNA expression in the respective modules was estimated by module signature (MS). Then correlation analysis between MS and clinical characteristics was performed. Co-expression modules with an absolute R value of > 0.6 were selected for further study (Fig. 2b). We found that the turquoise module was best correlated with PMT score, with an R value close to 0.9. Additionally, we found that the brown, yellow, and black modules were highly negatively related to PMT scores. All four above-mentioned modules were tightly associated with high-risk clinical and molecular features, such as WHO grade, Histopathology, molecular classification, TCGA subtype, Chr 7 gain/Chr 10 loss status, IDH status, and 1p/19q status. Thus, the turquoise, brown, yellow, and black modules were identified as modules of interest for further analyses.

Next, we screened core lncRNAs associated with PMT by setting the GS threshold to > 0.5 and the MM threshold to > 0.7 (Fig. 2c). This analysis identified 149 lncRNAs that were closely related to PMT (Fig. 2d and Additional file 1: Table S2). As expected, a strong relationship between several of these lncRNAs and mesenchymal transition in solid tumors had been reported in the literature. For example, mesenchymal transition was reinforced by CRNDE via the Wnt/β-catenin pathway in osteosarcoma [18], and lncRNA PVT1 sponged miR-195 to enhance EMT and induce therapeutic resistance in cervical cancer [19]. Therefore, we identified 149 lncRNAs that might play important roles in regulating mesenchymal transition.

### Establishing a PMT-related 10-lncRNA risk signature

**Fig. 3:**
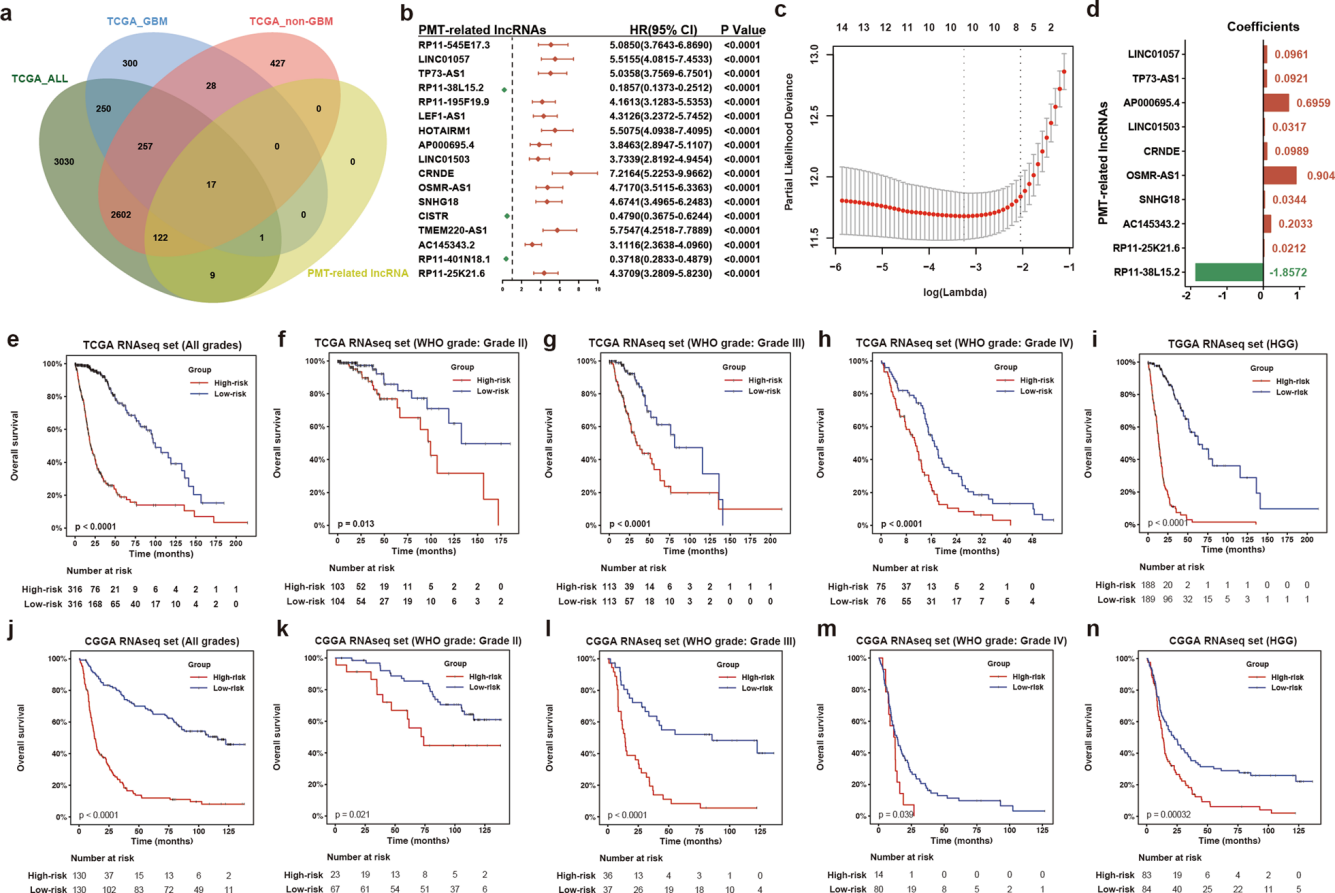
Construction of the PMT-related lncRNA risk signature. **a** The Venn diagram showed the PMT-related lncRNAs among prognosis-related lncRNAs in the all glioma, non-GBM, and GBM cohorts of TCGA RNAseq set. **b** Results of 17 prognosis-related lncRNAs from univariate Cox regression analysis in all cases from TCGA RNAseq set were shown in a forest plot. **c**, **d** LASSO analysis identified 10 lncRNAs that were used to construct a risk signature, and the corresponding coefficients were calculated. **e**–**n** The PMT-related 10-lncRNA risk signature exhibited powerful prognostic value in all, each grade or HHG from TCGA and CGGA datasets. *HHG* high-grade glioma

To further explore the prognostic value of these PMT-related core lncRNAs in glioma, we performed univariate Cox analyses on these lncRNAs in all glioma, non-GBM, and GBM cohorts in TCGA RNAseq set. This yielded 17 lncRNAs with significant prognostic value (P < 0.05) in the three cohorts that were selected to generate the lncRNA risk signature (Fig. 3a, b). The lncRNAs were of two types: i.e., protective and risky; 14 lncRNAs with HR > 1 were defined as risky, whereas three lncRNAs with HR < 1 were defined as protective.

To predict the prognosis of glioma cases by PMT-related lncRNAs, we used TCGA RNAseq set as the training dataset to perform LASSO regression analysis on these 17 lncRNAs. Finally, 10 of the lncRNAs were selected to construct a risk signature (Fig. 3c). Meanwhile, we used coefficients obtained from LASSO analysis to calculate risk scores for each of the TCGA and CGGA glioma cases (Fig. 3d). To analyze the prognostic value of the PMT-related 10-lncRNA risk signature, glioma cases in TCGA and CGGA RNAseq sets were dichotomized based on the median risk score. There was a significant difference in survival between the low- and high-risk groups (Fig. 3e, j). Stratified analyses of TCGA and CGGA datasets showed that higher risk scores were associated with unfavorable prognosis in all WHO grades (Fig. 3f–i, k–n). In summary, we established a PMT-related lncRNA signature with robust prognostic value in glioma.

### The risk signature was closely related to clinical and molecular features in glioma

**Fig. 4:**
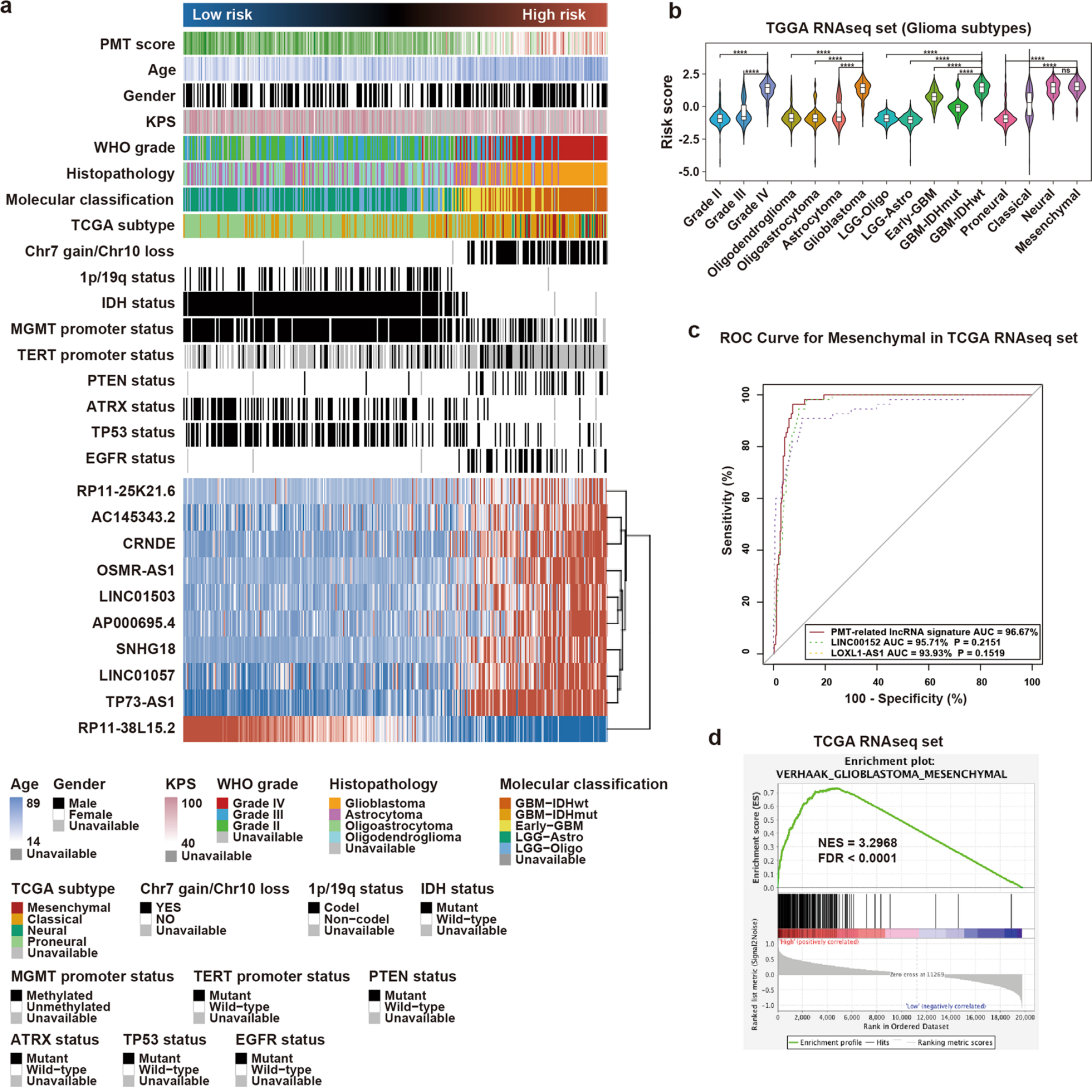
Correlation between the PMT-related risk signature and clinical and molecular features in TCGA. **a** The distribution of clinicopathological and molecular characteristics associated with the PMT-related 10-lncRNA signature in ascending order of risk score. **b** Distinct distributions of risk scores were seen among crucial factors, including WHO grade, histopathological type, molecular classification, and TCGA subtype. ****P < 0.0001; ns, non-significant. **c** Predictive value of the risk signature for the mesenchymal (MES) subtype was perform by ROC curve analysis in TCGA dataset. **d** Enrichment of the MES subtype in cases with high risk scores was verified by GSEA analysis in TCGA dataset

The heatmaps in Fig. 4a and Additional file 2: Fig. S4A showed the distribution of clinical and molecular features according to risk score. There was a significantly different distribution in age, Karnofsky performance score (KPS), WHO grade, histopathology, molecular subtype, TCGA subtype, Chr 7 gain/Chr 10 loss status, 1p/19q status, and related key molecular events (IDH, MGMT promoter, TERT promoter, PTEN, ATRX, TP53, and EGFR) between the high- and low-risk groups (Fig. 4a, Additional file 1: Table S3, Additional file 2: Fig. S4A). We also analyzed the correlation between risk score and clinical, pathological, and molecular characteristics. Cases with risky clinical factors and genomic events (MGMT promoter unmethylated, wild-type IDH, and 1p/19q non-codeletion) had higher risk scores (Fig. 4b, Additional file 2: Fig. S4B–G). Receiver operating characteristic curve (ROC) analyses indicated that risk signature might effectively predict the MES subtype in glioma. The areas under the corresponding ROC curves in TCGA and CGGA were 96.67% and 94.32%, respectively, which were both more than the areas of LINC00152 and LOXL1-AS1, and which were previously reported to be associated with PMT in glioma (Fig. 4c, Additional file 2: Fig. S4H). Furthermore, GSEA confirmed that glioma cases with high risk scores were more likely to be the MES subtype, whereas cases with low risk scores were more often the PN subtype (Fig. 4d, Additional file 2: Fig. S4I and J), indicating that the lncRNA risk score could discriminate PMT status. Additionally, we found that three (TP73-AS1, LINC01503, and CRNDE) of ten PMT-related lncRNAs highly expressed both in TCGA (Additional file 2: Fig. S9A) and CGGA (Additional file 2: Fig. S9B). TP73-AS1 and CRNDE were reported to promote malignant progression of glioma [20–23]. However, researches about the impact of LINC01503 on glioma were rare. Here, we selected LINC01503 as an example to validate the impact of lncRNAs on regulating PMT in glioma cell. In TCGA and CGGA, we found the expression level of LINC01503 was positively correlated to the mesenchymal marker (CD44; Additional file 2: Fig. S9C), but negatively associated with the pro-neural marker (OLIG2; Additional file 2: Fig. S9C). In U87 glioma cell, suppression of LICN01503 could significantly inhibit the expression of CD44, but increase OLIG2 expression, suggestive of mesenchymal-to-proneural transition (Additional file 2: Fig. S9D–F). Together, these findings showed the lncRNA risk signature was positively correlated with risky behaviors in glioma.

### Prognostic value of the risk signature in different stratified cohorts of high-grade glioma cases

**Fig. 5:**
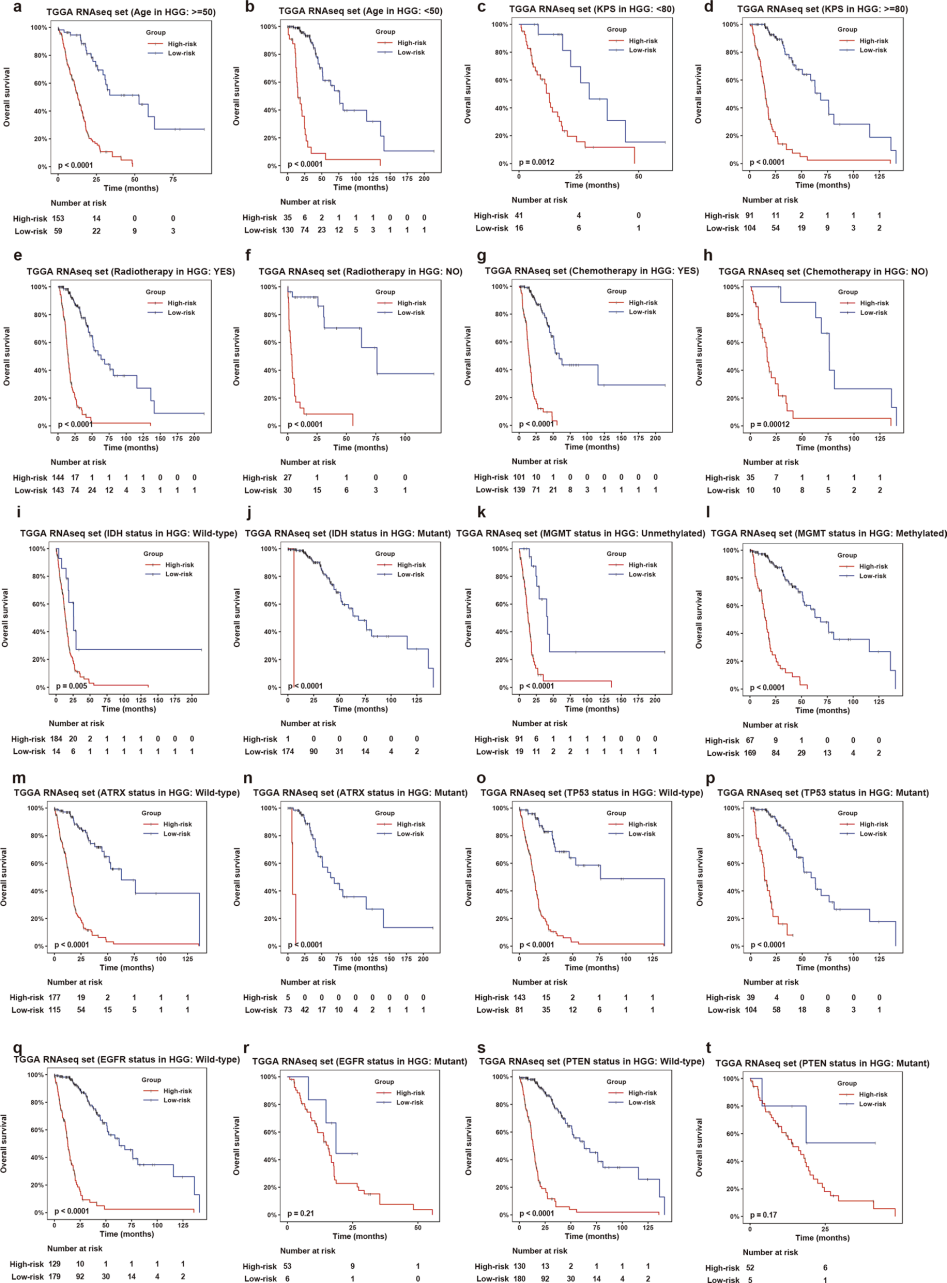
The prognostic value of the PMT-related 10-lncRNA risk signature in stratified groups of high-grade cases. **a**–**h** The risk signature showed significant prognostic value in different cohorts stratified by clinical features in TCGA dataset. **i**–**t** Prognostic value of the risk signature was most significant in subgroups stratified by key molecular events in TCGA dataset. The p-values were computed using the log-rank test for trend

The prognosis of high-grade (Grade III/IV) glioma cases is poor. To accurately evaluate the prognosis of these cases, we analyzed the prognostic value of the PMT-related 10-lncRNA risk signature in different stratified cohorts of high-grade glioma cases. In TCGA, we found that the risk score had significant predictive value in cohorts stratified by age and KPS (Fig. 5a–d). There was also a significant difference in survival time between the high- and low-risk groups regardless of whether the patients received radiotherapy or chemotherapy (Fig. 5e–h).

**Fig. 6:**
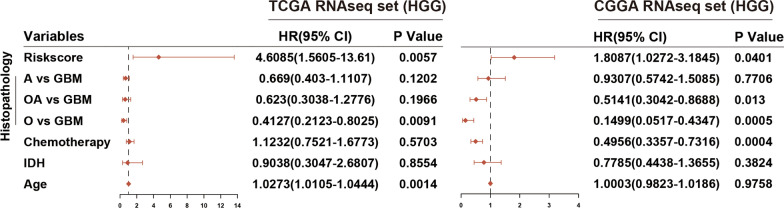
Multivariate Cox regression analysis of the risk signature. Forest plot showed the independent prognostic value of the risk signature compared with histopathologic classification, chemotherapy, IDH status, and age in TCGA and CGGA datasets. *HGG* high-grade glioma

Next, the prognostic value of the risk score in different molecular stratified cohorts was assessed. Under different statuses, such as IDH, MGMT promoter, ATRX, and TP53, cases with high risk scores had decreased survival time compared with cases with low risk scores (Fig. 5i–p). High scores indicated reduced OS in cases with wild-type EGFR and PTEN, but not in cases with mutant EGFR and PTEN (Fig. 5q–t). Similar results were validated in the CGGA dataset (Additional file 2: Fig. S5). Additionally, multivariate Cox analysis suggested that the risk signature was an independent prognostic factor in high-grade glioma (Fig. 6). In summary, the risk signature could significantly predict the prognosis of high-grade glioma.

### Biological functions of lncRNA in the risk signature

**Fig. 7:**
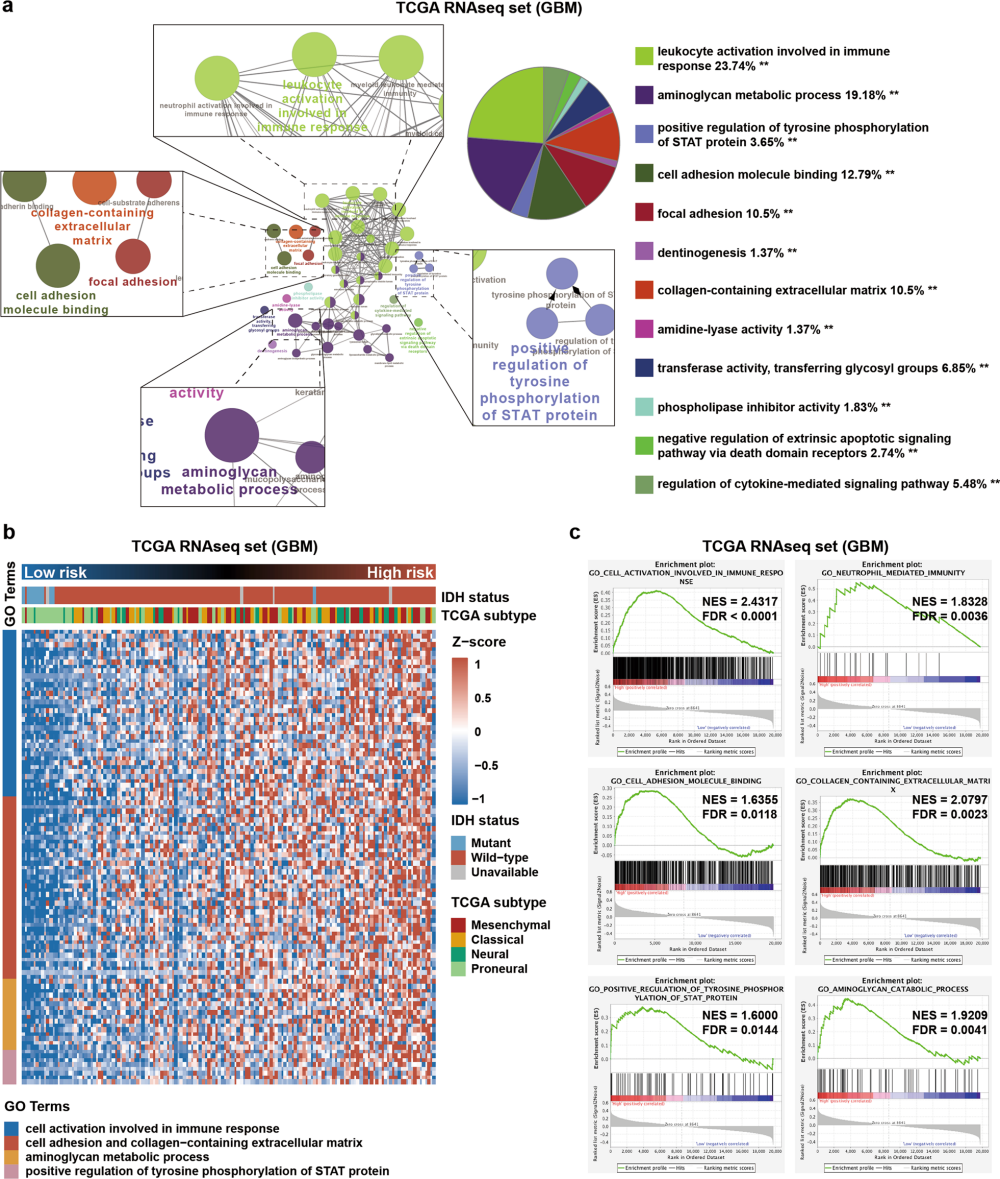
Functional exploration of the risk signature in GBM. **a** Gene ontology (GO) analysis using the ClueGO plugin in Cytoscape software was performed for coding genes positively correlated with risk score in TCGA. **P < 0.01. **b** Expression profiles of genes enriched in related GO terms were shown in the heat map. **c** Relative biological functions of these genes were verified by GSEA analyses

To explore the different functional features of GBM cases between the high- and low-risk groups, we first screened the coding genes that were positively correlated with the risk score (R > 0.3, p < 0.05) in GBM cases in the training dataset (TCGA, Additional file 1: Table S4). We then used the ClueGO plugin of Cytoscape to functionally annotate these coding genes. Then, we found that the most relevant biological processes in high-risk group were cell activation involved in immune response, cell adhesion and collagen-containing extracellular matrix, aminoglycan metabolic process, and positive regulation of tyrosine phosphorylation of STAT protein (Fig. 7a, b). These functional enrichments in the high-risk group were further verified by GSEA analyses (Fig. 7c). Analyses of the CGGA dataset revealed similar results (Additional file 1: Table S4; Additional file 2: Fig. S6). Additionally, previous studies have shown that STAT3 activation was positively associated with irradiation-induced PMT [24]. Taken together, we inferred that the 10 lncRNAs of the signature might facilitate biological processes that regulated PMT in GBM.

### GBM cases with high risk scores exhibited enhanced immunosuppression

**Fig. 8:**
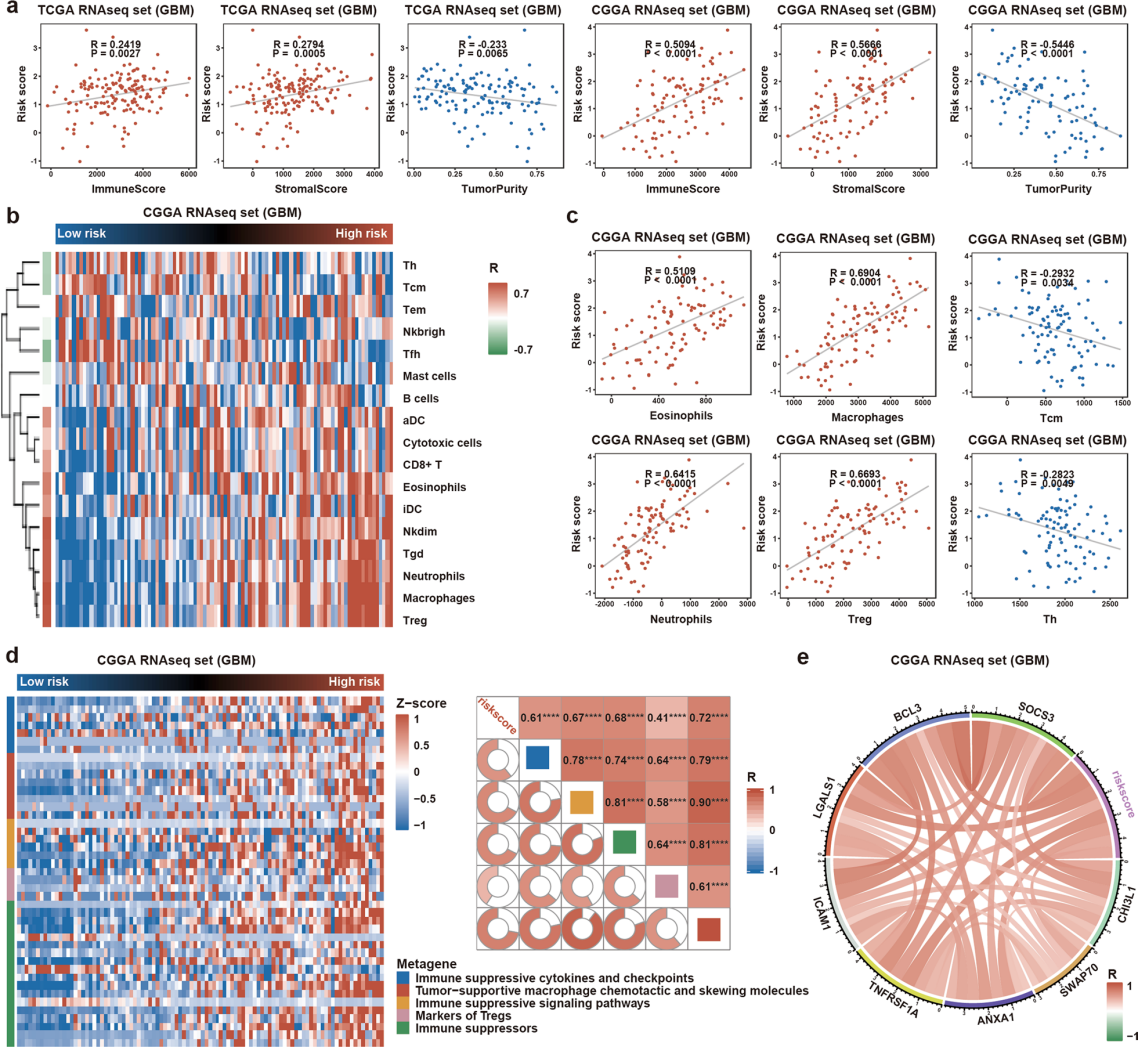
GBM cases with high risk scores exhibited enhanced immunosuppression. **a** Risk scores were positively associated with stromal scores and immune scores, whereas they were negatively correlated with tumor purity in TCGA and CGGA datasets. **b**, **c** Effect of the risk signature on the enrichment of immune cells. Risk scores were positively correlated with eosinophils, macrophages, neutrophils, and regulatory T cells, but negatively correlated with central memory T cells (Tcm) and helper T cells (Th) in the CGGA. **d** Risk scores were significantly positively correlated with immunosuppressive gene set scores in the CGGA. ****P < 0.0001. **e** The circle plot showed the relation between risk score and eight immunosuppressive effectors in CGGA

Recently, the MES subtype was reported to recruit more immunosuppressive cells than the PN and CL subtypes [25]. LncRNAs are important regulators of PMT, as described above; however, the correlation between PMT-related lncRNAs and immune responses in glioma are unknown. Therefore, we further analyzed immune responses related to the PMT-related lncRNA risk signature in GBM by calculating the scores of different immune cell subsets [26] by ssGSEA in the GSVA package, and immune score, stromal score, and tumor purity by the ESIMATE package [27] in GBM. Pearson’s correlation analysis was performed to evaluate the relationship between these tumor microenvironment indicators and risk score. We found that risk score was positively correlated with immune score and stromal score, but negatively correlated with tumor purity (Fig. 8a). Meanwhile, enrichment of eosinophils, macrophages, neutrophils, and regulatory T cells was positively associated with risk score, while central memory T cells and helper T cells were inversely related to risk score (Fig. 8b, c, Additional file 2: Fig. S7A and B). Further analyses showed that the PMT-related lncRNA risk score was positively related to the expression levels of the immunosuppressive genes described previously [28] (markers of Tregs, immunosuppressive signaling pathways, tumor-supportive macrophage chemotactic and skewing molecules, and immune suppressors; Fig. 8d, Additional file 2: Fig. S7C and D). Next, we intersected RSGs from TCGA and CGGA with immunosuppressive genes (Additional file 2: Fig. S7E). Finally, eight effectors were identified to be significantly positively correlated with the risk signature (Fig. 8e, Additional file 2: Fig. S7F). Together, these findings indicated that the ten lncRNAs could reinforce PMT to enrich certain immune cell subsets in the tumor microenvironment.

### Constructing the PMT-related lncRNA/miRNA/mRNA network

Previous studies have shown that lncRNAs can act as ceRNAs to sponge miRNAs and release the coding mRNA to enhance its biological function. To explore whether any of the 10 PMT-related lncRNAs acted as ceRNAs, we predicted the potential miRNAs that could bind these lncRNAs using the DIANA website and intersected them with the downregulated miRNAs in GBM. Then, 15 miRNAs were selected for subsequent analyses. The potential target genes of these miRNAs were predicted by the starBase v3.0 database. Next, these target genes were intersected with the coding genes positively related to risk score in both TCGA and CGGA. In total, 82 potential target genes were identified (Additional file 2: Fig. S8A).

Subsequently, we used Cytoscape to build an initial ceRNA network based on the filtered lncRNAs, miRNAs, and mRNAs (Additional file 2: Fig. S8B). Meanwhile, we constructed a protein interaction network to analyze relationships among the 82 target genes. Then, nine core genes were screened by the MCODE method, with k = 2 and degree = 3 (Additional file 2: Fig. S8C). We further generated a core lncRNA/miRNA/hub gene network that included six lncRNAs, 10 miRNAs, and nine core coding mRNAs (Additional file 2: Fig. S8D). Further GO analyses by ClueGO showed that the functions of these nine core genes were involved in regulating leukocyte aggregation, and positive regulation of adhesion organization and regulation of apoptosis (Additional file 2: Fig. S8D). Moreover, some of the ceRNAs in the network had previously been reported to affect risky behaviors and immune responses. For example, miR-512-3p binded to CD44, resulting in suppression of invasion and cell adhesion in breast carcinoma [29]. Additionally, the mesenchymal marker CD44 was identified to significantly positively regulate PD-L1 expression in breast cancer and non-small cell lung cancer [30]. Overall, these results suggested that several PMT-related lncRNAs could promote mesenchymal transition and elicit immunosuppression via this ceRNA network in GBM.

## Discussion

Verhaak et al. [3] used TCGA core gene expression profiles to classify GBM into four subtypes: PN, NL, CL, and MES. This classification system is also applicable for non-GBM. PN primarily exists in grade II/III gliomas, while CL and MES are mainly found in GBM [31]. In the study by Verhaak et al. there was no significant difference in survival time among the different subtypes [3]. However, recent studies have shown that PN cases have a good prognosis, NL cases have intermediate prognosis, whereas CL and MES cases display unfavorable outcomes [6]. Recently, individualized therapeutic strategies in gliomas were significantly improved by the molecular classification of glioma patients; however, the transition from PN to MES, which is induced by radiotherapy or chemotherapy, is a major trigger for treatment resistance [32]. Therefore, PMT status should be taken into account when developing an individualized treatment course. To date, there are no comprehensive methods of evaluating PMT status, meanwhile, indicators that drive PMT in glioma remain obscure. In this study, we used ssGSEA to calculate PMT scores, which could successfully estimate the PMT status in glioma. We found that PMT score was closely related to clinical and molecular characteristics in glioma. PMT scores were higher in cases with risky phenotypes, specifically up-regulated in IDH wild-type and MES subtype, which were recognized as subgroups with strong invasiveness and enhanced immune response [33, 34]. Meanwhile, we also found that PMT score could predict the prognosis of glioma cases, even in GBM. Additionally, chemotherapy was more effective in cases with low PMT scores, whereas cases with high PMT scores showed less therapeutic efficacy, confirming the importance of PMT on clinical outcomes.

PMT scores were calculated from PN and MES gene sets. Although PMT score had significant prognostic value, too many effectors were included, limiting its ability for clinical translation. Recent studies have shown that lncRNAs promote mesenchymal transition in glioma. For example, H19 enhances Sox4 expression by sponging miR-130a-3p, which promotes EMT in glioma [35]. Additionally, LINC00152 regulates PMT in glioma via the miR-612/AKT2/NF-kB axis [10]. In this study, we used the WGCNA algorithm [36], which is a powerful method of identifying candidate biomarkers or therapeutic targets, to screen lncRNA co-expression modules closely related to PMT score, and identified 10 core lncRNAs associated with prognosis. The PMT-related 10-lncRNA risk signature also had powerful predictive value. Similar to PMT scores, risk scores were positively correlated with risky behaviors and had significant predictive value for PMT status in glioma. Furthermore, a signature consisting of only 10 lncRNAs is more suitable for clinical application.

Among these 10 lncRNAs, nine (LINC01057, TP73-AS1, AP000695.4, LINC01503, CRNDE, OSMR-AS1, SNHG18, AC145343.2, and RP11-25K21.6) were high-risk lncRNAs, while only one (RP11-38L15.2) was a protective lncRNA. Several previous studies have revealed that some of these lncRNAs participate in solid tumor risky progression. For example, high TP73-AS1 expression is associated with poor prognosis, and inhibiting TP73-AS1 expression suppresses EMT in gastric cancer and bladder cancer [37, 38]. Overexpressing CRNDE promotes EMT in oral epithelial cell carcinoma and osteosarcoma [39, 40]. In glioma, high SNHG18 expression enhances radiation resistance, and triggers EMT by increasing ENO1 expression [41]. To the best of our knowledge, no studies have investigated the effects of these lncRNAs on PMT in glioma. Here, we further explored the biological functions of the PMT-related lncRNA signature. Most of the biological functions of the high-risk group were enriched in activation of immune response, regulation of cell adhesion, enhanced amino acid and glucose metabolism, and activation of STAT family genes, which are biological characteristics of the MES subtype. Moreover, previous studies have shown that inhibiting STAT3 activation suppresses radiation-induced PMT in glioma [24]. Additionally, we proved that LINC01503, one of these ten lncRNAs, could promote PMT in glioma through biological experiments. Together, these results suggest that this 10-lncRNA signature could contain potential molecular targets for glioma treatment.

The immune cells enriched in glioma play a crucial role in malignant progression and recurrence. Compared with PN, MES exhibited enhanced immunosuppression by being enriched for more tumor-associated macrophages (TAMs), CD3+, and FOXP3+ T cells [25]. Thus, the shift from PN to MES in glioma might reprogram the immunological microenvironment, resulting in enhanced tumor immunosuppression. These findings indicate that reversing PMT could improve the clinical outcomes from immunotherapy in glioma. Here, we found that the PMT-related lncRNA signature significantly reflected immune status in GBM. Risk scores were positively correlated with enrichment of eosinophils, macrophages, neutrophils, and Tregs in the glioma microenvironment. TAMs are the most abundant immune cells in glioma and create favorable conditions for growth and tumor cell invasion, resulting in unfavorable prognosis. Furthermore, we found that risk scores were positively correlated with immunosuppressive effectors, including CHI3L1, SWAP70, ANXA1, TNFRSF1A, ICAM1, LGALS1, BCL3, and SOCS3. Recent studies have shown that these immunosuppressive effectors enhance tumor immunosuppression. For example, CHI3L1 is secreted by fibroblasts and reshapes the breast cancer microenvironment by promoting the enrichment of M2 macrophages [42]. Mesenchymal stem cells can acquire immunosuppressive capacity by gaining ICAM1 expression [43]. TNFRSF1A mediates STAT3 phosphorylation and promotes the accumulation of myeloid suppressor cells in the tumor microenvironment [44]. SWAP70 restricts the maturation of dendritic cells [45]. However, very little is known about correlations between the 10 lncRNAs and eight immunosuppressive genes. Therefore, further experiments are needed to verify the mechanisms of these lncRNAs on reprogramming the immunosuppressive microenvironment in glioma.

Prior studies have noted that lncRNAs carry abundant miRNA binding sites, which could sponge miRNAs to indirectly enhance the expression of downstream target genes [46–48]. In this study, we explored whether the 10 lncRNAs in the PMT signature affected risky behaviors and immune responses via such indirect mechanisms. Finally, a core lncRNA/miRNA/mRNA network was established, which included six lncRNAs (AC145343.2, SNHG18, OSMR-AS1, AP000695.4, TP73-AS1, and LINC01057), ten miRNAs (hsa-miR-103a-3p, hsa-miR-107, hsa-miR-128-3p, hsa-miR-129-5p, hsa-miR-338-3p, hsa-miR-381-3p, hsa-miR-410-3p, hsa-miR-491-5p, hsa-miR-495-3p, and hsa-miR-512-3p), and nine core coding mRNAs (IQGAP1, CASP8, BIRC3, RAC1, MSN, ANXA5, ICAM1, CD44, and TNFRSF1A). In reviewing the literature, TP73-AS1 was found to combine with miR-124 and miR-142, resulting in enhanced proliferation of glioma cells [22, 49]. Additionally, BIRC3, ICAM1, and TNFRSF1A belong to the group of immunosuppressive factors described above, and CD44 is a core MES marker [4]. Furthermore, a previous study showed that CD44 expression was significantly inhibited by miR-512-3p in breast cancer [29]. Additionally, OPN, a physiological ligand of CD44, can bind to CD44 and activate T cells [50]. Most recently, CD44 was identified to significantly increase PD-L1 expression, enhancing immunosuppression by alleviating T cell enrichment in breast cancer and lung cancer [30]. According to these findings, we inferred that several PMT-related lncRNAs could indirectly promote PMT and reinforce the immunosuppressive status of GBM through a ceRNA network.

## Conclusions

In summary, we explored the PMT status of glioma and identified 10 PMT-related lncRNAs in a large sample size of glioma cases. Our newly developed risk signature was constructed on the basis of these lncRNAs and could successfully evaluate PMT status. Thus, we believe that it could serve as a clinical prognostic indicator for high-grade glioma. Meanwhile, several of the identified PMT-related lncRNAs could act as ceRNAs to trigger PMT, resulting in enhanced immunosuppression in GBM. However, the main weakness of this study was that major conclusions came from retrospective analyses of large public datasets. Further research should be undertaken to verify the biological functions and mechanism of these lncRNAs in PMT, which will provide new insights into individualized therapeutic strategies.

## Supplementary information

**Additional file 1. Table S1.** Clinical and molecular information of glioma cases included in this study.** Table S2.** 149 PMT-related lncRNAs in four modules.** Table S3.** The distribution of clinical and molecular charateristics between high- and low-risk groups.** Table S4.** Genes positively correlated with risk score in GBM in TCGA and CGGA.

**Additional file 2: Figure S1.** Distribution and prognostic value of PMT scores in gliomas from multiple datasets. **Figure S2.** Influence of PMT score on responses to chemotherapy or radiotherapy. **Figure S3.** Evaluation of the distribution of glioma cases in a scale-free network. **Figure S4.** Associations between the PMT-related risk signature and other features in TCGA and CGGA datasets. **Figure S5.** The predictive value of the risk signature on survival was verified in high-grade cases from the CGGA. **Figure S6.** Functional enrichment of the risk signature was verified in the CGGA. **Figure S7.** Correlations between immunosuppressive status and the risk signature in GBM. **Figure S8.** Construction of the PMT-related lncRNA/miRNA/mRNA (ceRNA) network in GBM. **Figure S9.** The effect of LINC01503 on PMT in glioma cell.

## Data Availability

Please contact author for data requests.

## Abbreviations

lncRNAs: Long non-coding RNAs
miRNA: MicroRNA
mRNA: Messenger RNA
TCGA: The Cancer Genome Atlas
CGGA: The Chinese Glioma Genome Atlas
GO: Gene ontology
GSEA: Gene set enrichment analysis
TANRIC: The atlas of noncoding RNAs in cancer
SRGs: Signature-related genes
logFC: Log2(fold-change)
PPI: Protein–protein interaction
MCODE: Molecular complex detection
OS: Overall survival
ceRNA: Competing endogenous RNA
PN: Pro-neural
NL: Neural
CL: Classical
MES: Mesenchymal
GBM: Glioblastoma
PMT: Pro-neural to mesenchymal transition
EMT: Epithelial to mesenchymal transition
ssGSEA: Single sample gene set enrichment analysis
WGCNA: Weighted gene co-expression network analysis
WHO: World Health Organization
KPS: Karnofsky performance score
GBM-IDHwt: GBM with wild-type IDH
HR: Hazard ratio
DELs: Differentially-expressed lncRNAs
