# Supplementary material for: Profiling pro-neural to mesenchymal transition identifies a lncRNA signature in glioma
Source: J Transl Med. 2020 Oct 7;18:378. doi: 10.1186/s12967-020-02552-0 (PMC7539462; doi:10.1186/s12967-020-02552-0)
Supplement: Supplementary file 2 — Additional file 2: Figure S1. Distribution and prognostic value of PMT scores in gliomas from multiple datasets. Figure S2. Influence of PMT score on responses to chemotherapy or radiotherapy. Figure S3. Evaluation of the distribution of glioma cases in a scale-free network. Figure S4. Associations between the PMT-related risk signature and other features in TCGA and CGGA datasets. Figure S5. The predictive value of the risk signature on survival was verified in high-grade cases from the CGGA. Figure S6. Functional enrichment of the risk signature was verified in the CGGA. Figure S7. Correlations between immunosuppressive status and the risk signature in GBM. Figure S8. Construction of the PMT-related lncRNA/miRNA/mRNA (ceRNA) network in GBM. Figure S9. The effect of LINC01503 on PMT in glioma cell. [file 12967_2020_2552_MOESM2_ESM.docx]

**Additional figures**

**
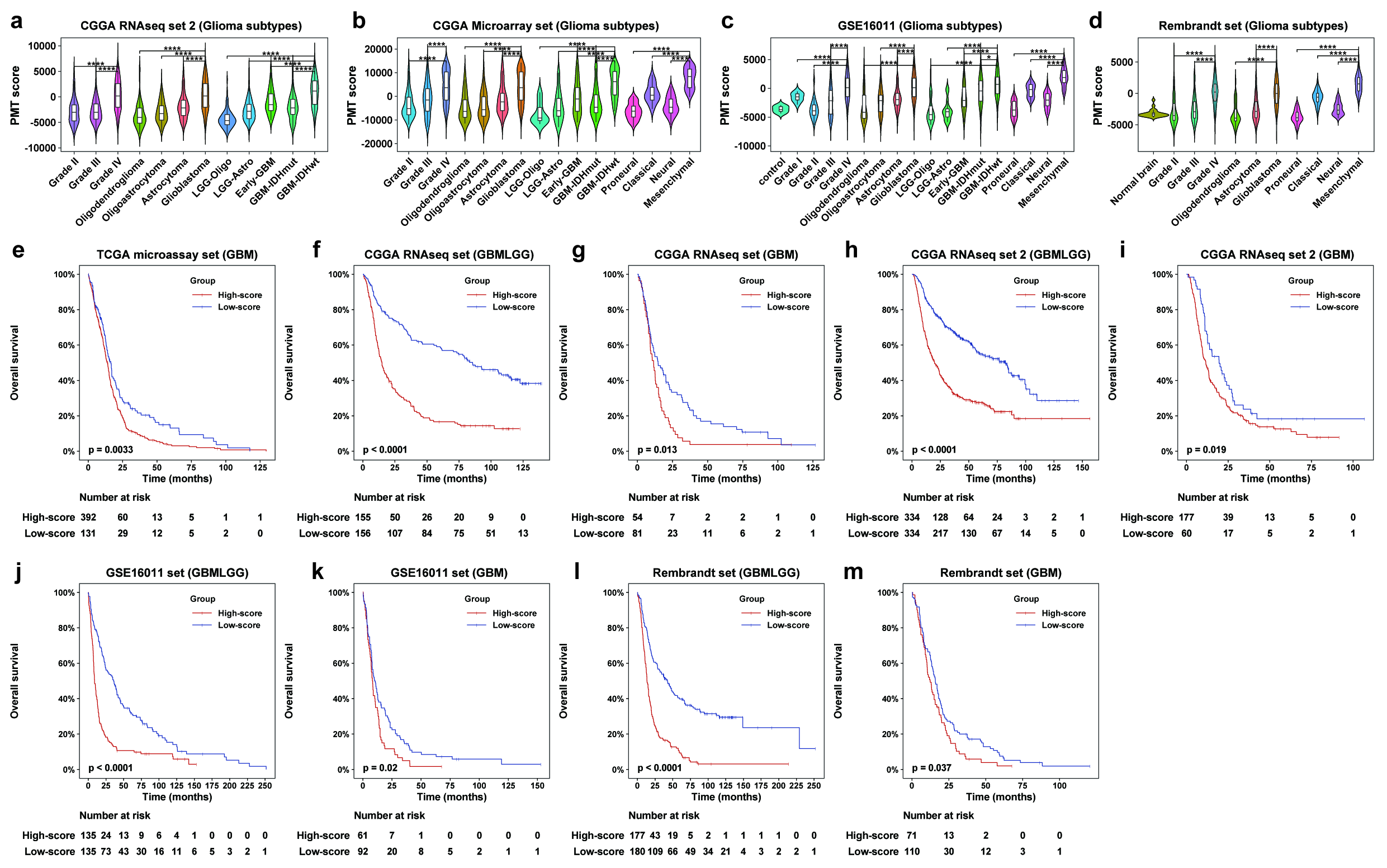
**

**Fig. S1 Distribution and prognostic value of PMT scores in gliomas from multiple datasets. (a-i)** Evaluation of the distribution and prognostic value of PMT scores across various glioma subtypes in CGGA datasets, GSE16011, and Rembrandt. ^*^p < 0.05; ^****^p < 0.0001.

**
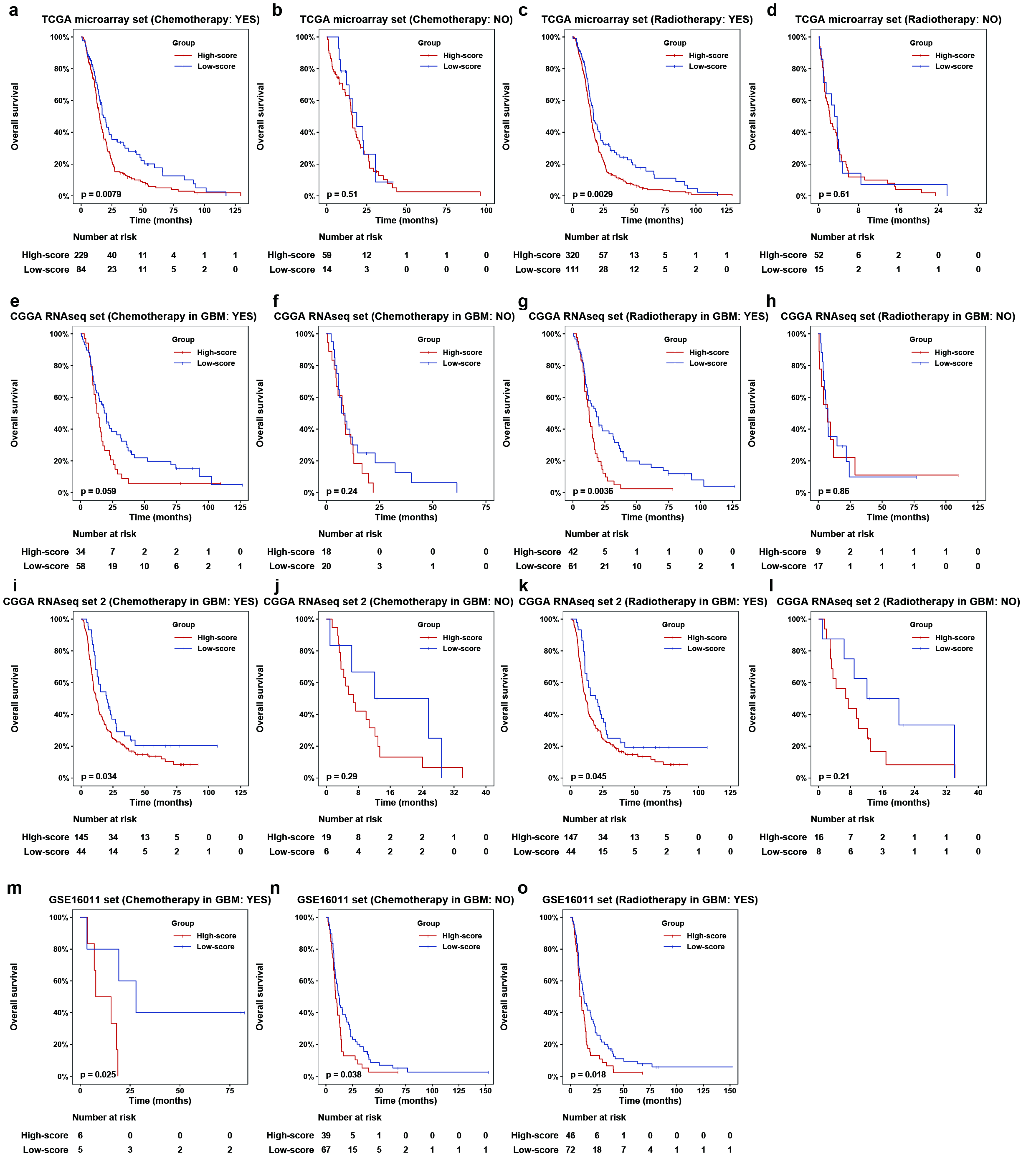
**

**Fig. S2 Influence of PMT score on responses to chemotherapy or radiotherapy. (a-o)** Prognostic value of PMT scores in GBM with or without chemotherapy or radiotherapy from four public glioma datasets. The p-values were computed using the log-rank test for trend.


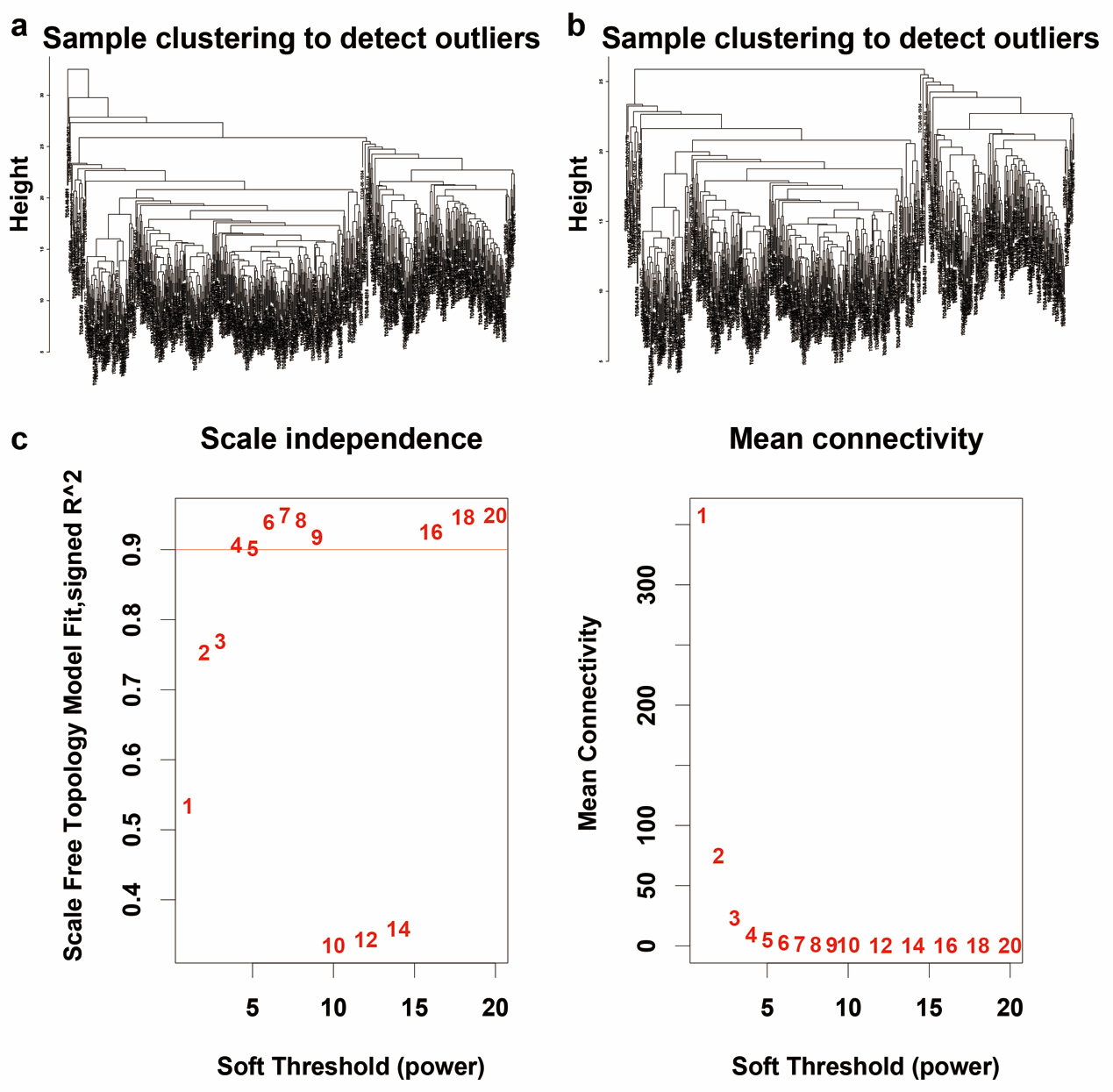


**Fig. S3 Evaluation of the distribution of glioma cases in a scale-free network. (a)** Clustering of cases before removing outliers. (**b)** Clustering of cases after removing outliers with cutHeight = 27. (**c)** The soft-thresholding value was analyzed for the scale-free network.


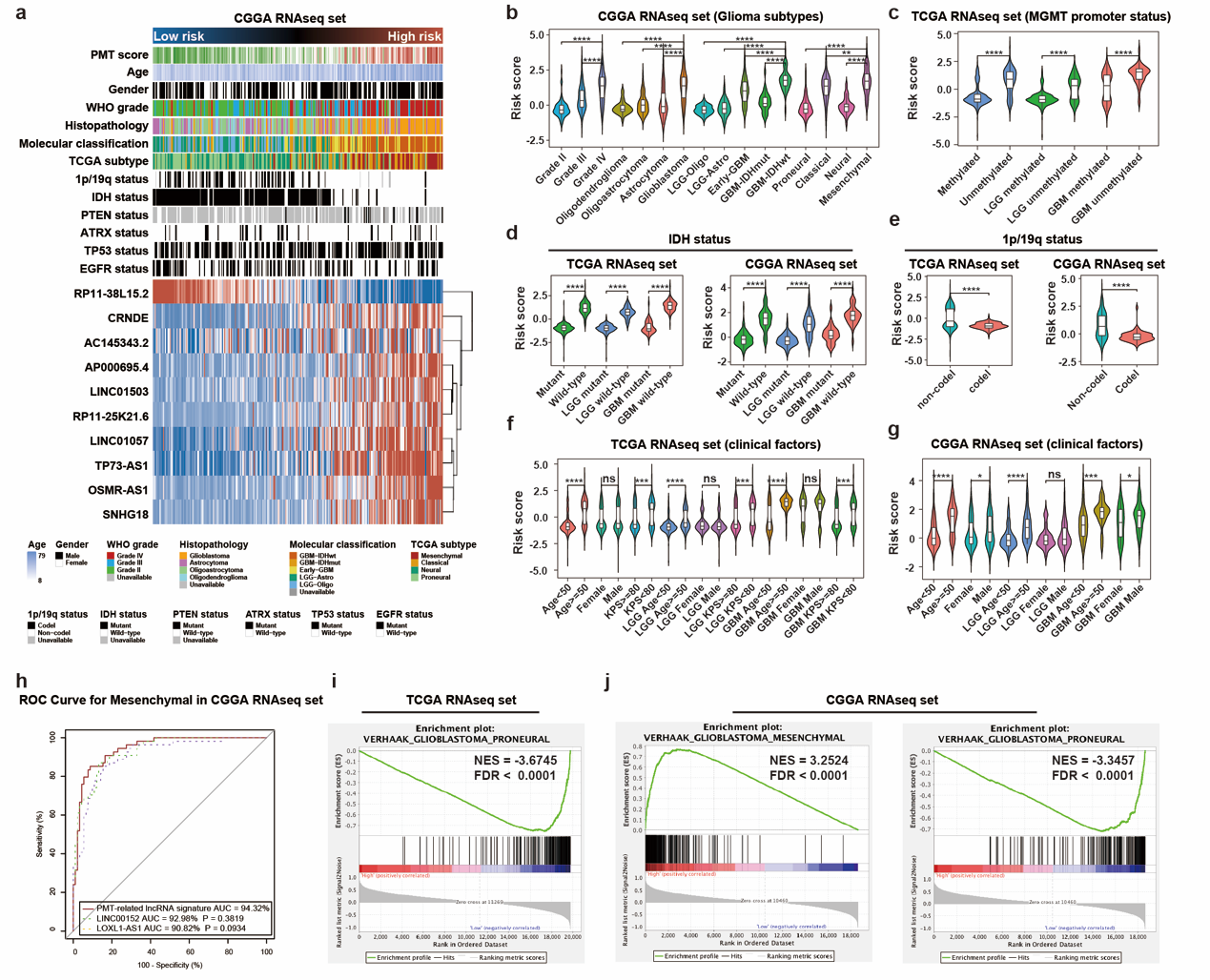


**Fig. S4 Associations between the PMT-related risk signature and other features in TCGA and CGGA datasets. (a)** Correlations among the risk signature and clinical and molecular features as well as related lncRNAs in the CGGA. (**b-g)** Risk scores exhibited distinct distribution patterns in various stratified subgroups from TCGA and CGGA datasets. (**h-j)** GSEA analyses showed enrichment of high-risk cases in the mesenchymal subtype, and low-risk cases in the pro-neural subtype. ^*^p < 0.05; ^**^p < 0.01; ^***^p < 0.001; ^****^p < 0.0001; ns, non-significant.


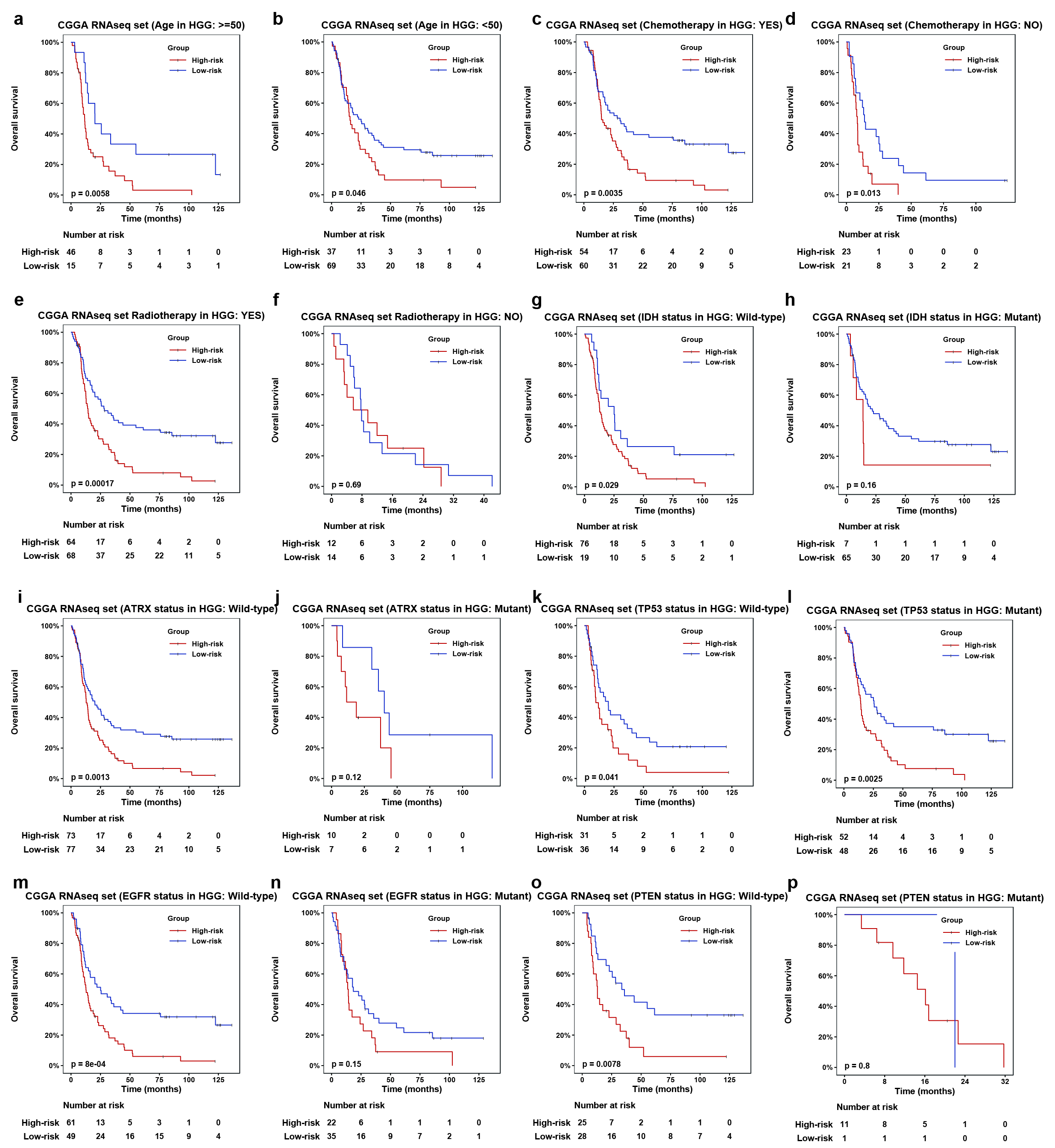


**Fig. S5 The predictive value of the risk signature on survival was verified in high-grade cases from the CGGA.** The p-values were computed using the log-rank test for trend.

**
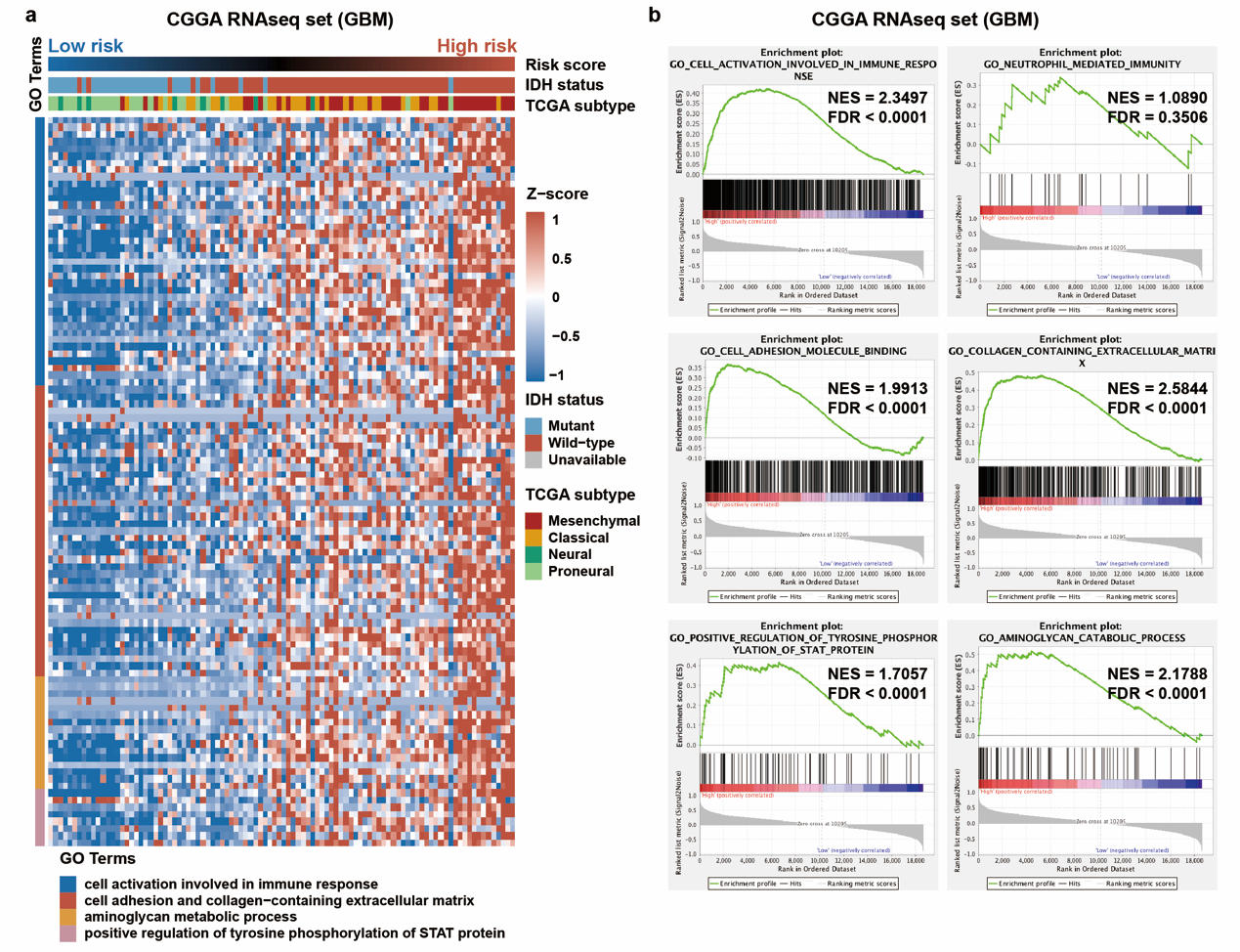
**

**Fig. S6 Functional enrichment of the risk signature was verified in the CGGA.**

**
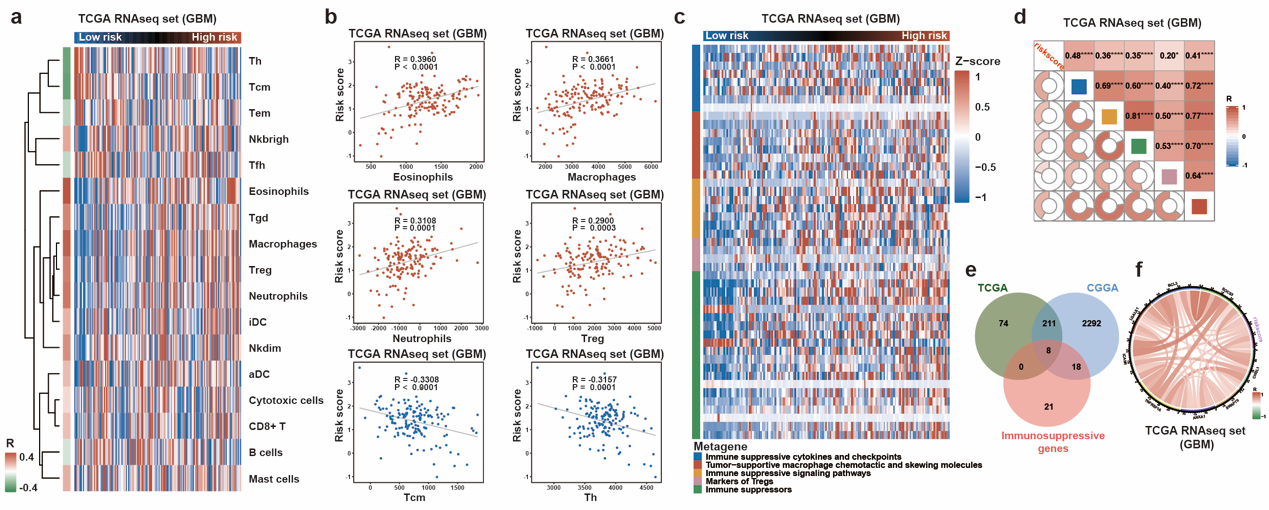
**

**Fig. S7 Correlations between immunosuppressive status and the risk signature in GBM.** (**a, b)** Heatmap and scatter plot showing the positive correlation of risk scores and immunosuppressive cells, including eosinophils, macrophages, neutrophils, and regulatory T cells in TCGA datasets. (**c, d)** Immunosuppressive scores calculated from related gene sets were significantly positively associated with the risk score in TCGA datasets. ^*^p < 0.05; ^****^p < 0.0001. (**e)** Venn diagram showing the intersection between immunosuppressive genes and genes positively correlated with risk score in both TCGA and CGGA datasets. (**f)** Circle plot depicting the correlation of risk score with the eight immunosuppressive genes in TCGA datasets.


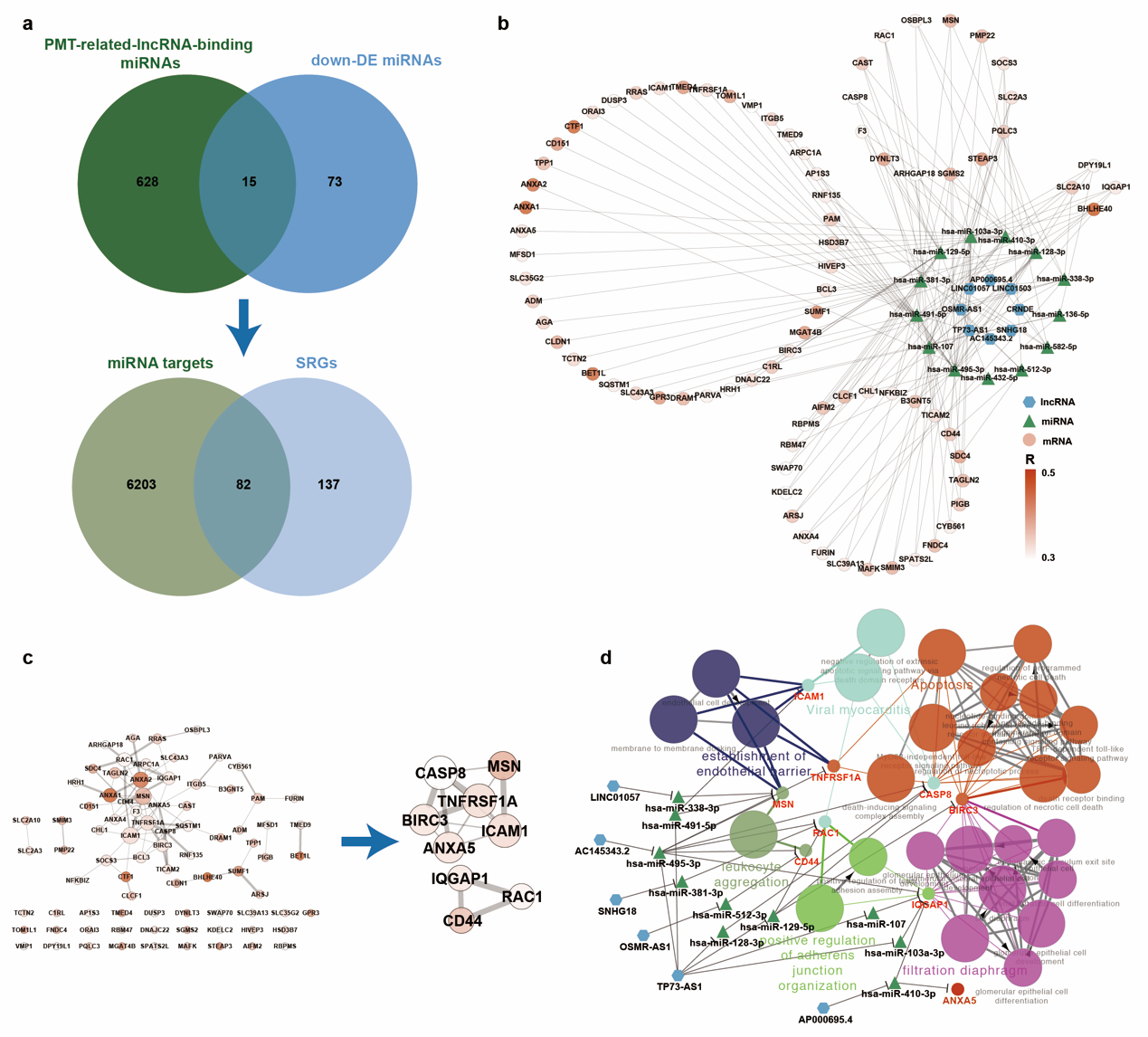


**Fig. S8 Construction of the PMT-related lncRNA/miRNA/mRNA (ceRNA) network in GBM. (a)** Venn diagram showing the miRNAs downregulated in GBM (down-DE miRNAs) among miRNAs predicted to interact with the PMT-related 10 lncRNAs, and predicted targets of the 15 miRNAs among the coding genes positively correlated with the risk signature (signature-related genes; SRGs). (**b)** An initial ceRNA network showed a correlation among corresponding lncRNAs, miRNAs, and coding genes. The correlation coefficient (R) represented the correlation between risk scores and expression levels of coding genes, which decreased in size from red to white. (**c)** A protein-protein interaction (PPI) network of 82 potential downstream targets of the eight PMT-related lncRNAs was constructed by the PPI function, and nine core targets were identified by MCODE in Cytoscape. (**d)** A core ceRNA network including six lncRNAs, ten miRNAs, and nine core targets was subsequently generated. The core targets were annotated by the ClueGO plugin. DE, differential expression, ceRNA, competing endogenous RNA.


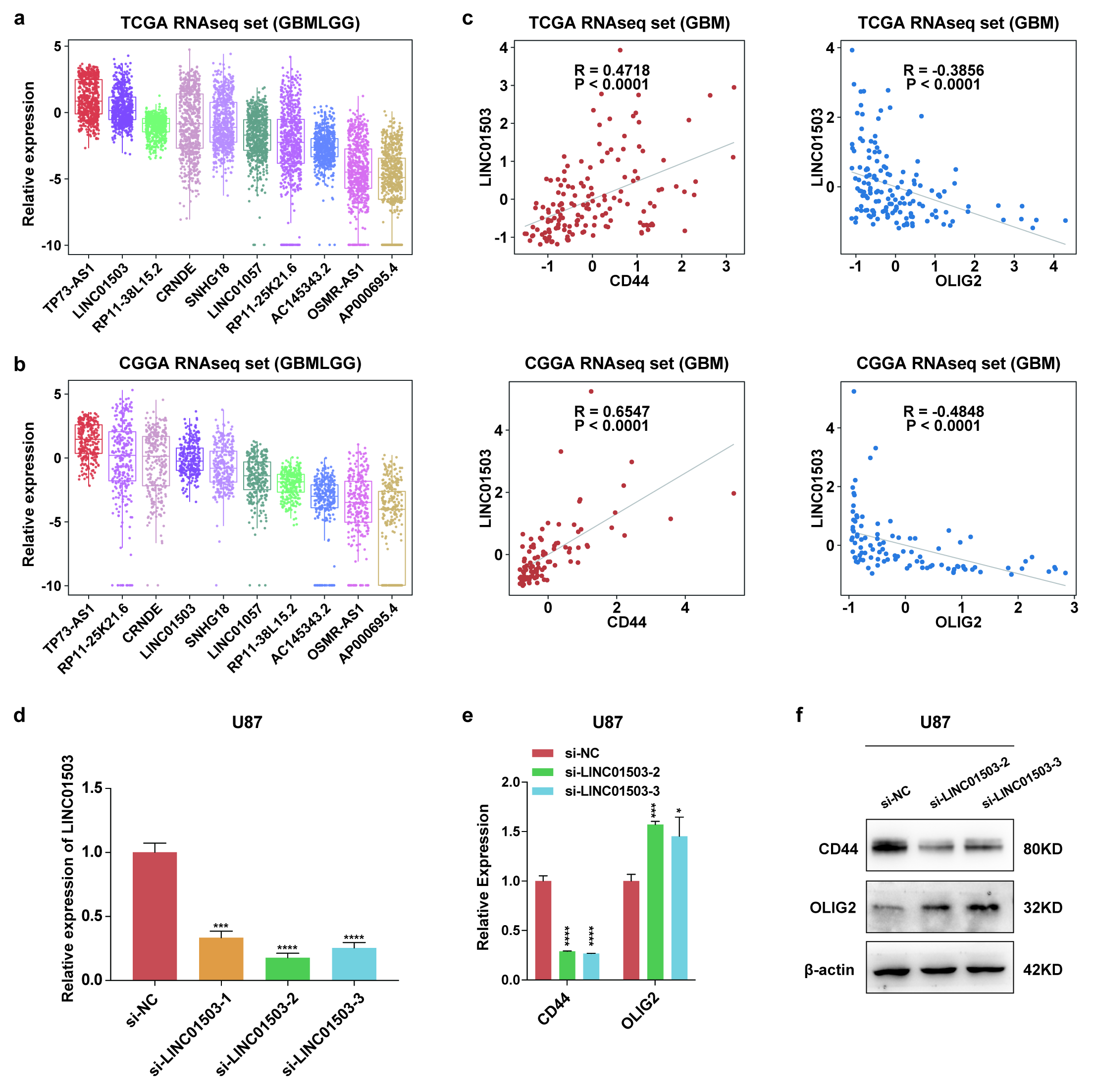


**Fig. S9 The effect of LINC01503 on PMT in glioma cell. (a, b)** The relative expression levels of ten PMT-related lncRNAs in TCGA and CGGA. **(c)** The relation of LINC01503 and PMT related markers (Mesenchymal marker, CD44; Pro-neural marker, OLIG2) in GBM of TCGA and CGGA. **(d)** qRT-PCR was performed to validate the efficiency of si-LINC01503-1, -2, and -3 in U87 glioma cell. **(e, f)** qRT-PCR and western blot analyses of CD44 and OLIG2 expression following knockdown of LINC01503 in U87; β-actin was used as a loading control. ^*^p < 0.05; ^***^p < 0.001; ^****^p < 0.0001.
